# Comparison of two advanced bipolar tissue sealer/dividers for laparoscopic ovariectomy in dogs: articulating enseal G2 versus Ligasure Maryland device

**DOI:** 10.1186/s13028-023-00715-9

**Published:** 2023-11-29

**Authors:** Floor Driessen, Javier Deniz Marrero, Guy Cornelis Maria Grinwis, Sebastiaan Alexander van Nimwegen

**Affiliations:** 1 Department of Clinical Sciences, Faculty of Veterinary Medicine, Utrecht University, Yalelaan 108, Utrecht, 3584 CM The Netherlands; 2 Department of Biomolecular Health Sciences, Faculty of Veterinary Medicine, Utrecht University, Yalelaan 1, Utrecht, 3584 CL The Netherlands

**Keywords:** Canine, Duration seal and cut, Histopathology, Laparoscopy, Minimal invasive, Neutering, Re-sterilization, Re-use, Thermal tissue damage

## Abstract

**Background:**

Advanced bipolar tissue sealer/dividers provide the most reliable and efficient means of tissue dissection and blood vessel sealing in laparoscopic surgery and the techniques are continuously improved. In veterinary practice, cost-effectiveness is of major impact, leading to re-use of instruments designed and sold for single use. Two high-end devices were evaluated and compared in a highly standardized laparoscopic ovariectomy procedure in dogs: The new generation Ligasure Maryland Sealer/Divider (LMSD) with improved atraumatic curved jaw shape for delicate tissue handling and dissection and non-stick nanocoating, and the new-generation Articulating Enseal G2 (AENG2) with several proclaimed features improving surgical performance, including articulation of the forceps tip; improved tissue compression during sealing; unique offset electrode configuration; and specific nanoparticle coating minimizing thermal spread and tissue sticking. Twenty-one client-owned dogs admitted for elective laparoscopic ovariectomy were randomly assigned to one of two groups: ovariectomy using AENG2 on the left ovary and LMSD in the right ovary or *vice-versa*. Procedural video recordings were used to assess ovarian ligament fat score, smoke formation, occurrence of bleeding, and excision duration. Excised tissues were examined histopathologically and collateral thermal damage was scored in three anatomic zones: suspensory ligament, vascular pedicle, and uterine junction. Tissue sealers were used repeatedly following standardized cleaning protocol with instrument washing machine and ethylene oxide gas sterilization and the number of uses until device failure was recorded.

**Results:**

Excision times were significantly increased for AENG2 (median 01:35 min) compared to LMSD (median 01:00 min). Minor hemorrhage from incomplete sealing occurred in 3 sites in 2 patients (2x AENG2, 1x LMSD) and was not significantly different between groups. Smoke production as scored on videos and thermal tissue damage scores on histopathology also did not differ between AENG2 and LMSD. Both vessel sealers could be re-used repeatedly.

**Conclusion:**

AENG2 provides a good alternative to LMSD in laparoscopic ovariectomy, with only minor differences in measured variables. Subjectively, the articulating feature of AENG2 did not improve surgical performance in laparoscopic ovariectomy and the use of LMSD appeared more straight-forward for this specific procedure. However, differences in operating these devices may be subject to personal preference.

## Background

Advanced tissue sealers have been vital in the development of minimally invasive surgery, improving surgical performance, reducing complications and procedural duration. LigaSure tissue sealer/divider has been considered to set a gold standard in laparoscopic surgery: the device-controlled tissue sealing technique is reliable, fast, easy to use, and has been shown effective to seal blood vessels up to 7 mm in diameter [1–10]. Enseal is another leading brand of advanced bipolar tissue sealer/dividers that has previously been shown to be a competitive option compared to LigaSure [11–14]. Older versions of both devices scored comparably high in terms of seal quality and thermal tissue damage extent while outperforming most other competitors [2, 6, 10, 15–17]. The new generation Ligasure Maryland Sealer/Divider (LMSD) provides an improved atraumatic curved jaw shape for delicate tissue handling and improved tissue dissection, with non-stick nanocoating technology [18]. Tissue sealing is completely device-controlled. The new-generation Articulating Enseal G2 (AENG2) has several proclaimed features to improve surgical performance and tissue sealing, including articulation of the forceps tip; a specifically designed jaw mechanism to improve uniform tissue compression during sealing; an offset electrode configuration and ‘positive temperature coefficient nanoparticles’ in the jaw to minimize thermal spread and tissue sticking [17, 19]. Completion of tissue sealing is partly user-controlled.

To date, direct comparison of LMSD and AENG2 have only been performed in total laparoscopic hysterectomy in humans. Compared to LMSD, AENG2 was associated with an increase in operation time [12, 14], an increase in surgeon-perceived workload, and a higher rate of device failure [14]. The use of Enseal has been described in dogs for sealing of pulmonary arteries and transection of vaginal septa [13, 20]. Although most advanced vessel sealers are developed for single-use they are commonly being re-used in veterinary practice for cost-effectiveness. Successful re-use of LigaSure devices after repeated washing/sterilization cycles has been described previously [5, 21–24]. To our knowledge, repeated use of the AENG2 after re-sterilization has not been described.

The aim of the present study was to evaluate and compare surgical performance, complications and thermal tissue damage in laparoscopic ovariectomy in dogs using (AENG2) *versus* (LMSD). Furthermore, the possibility of repeated use of each device was assessed after repeated cleaning and re-sterilization, to evaluate if devices would be of similar interest for veterinary practice. Based on published data in human hysterectomy, we hypothesized that there would be a difference in ovariectomy duration between LMSD and AENG2. Based on published data of older versions of both devices, we would not expect significant differences in bleeding, smoke formation, and thermal tissue damage extent.

## Methods

### Patient selection

Client-owned healthy dogs presented for elective laparoscopic ovariectomy at the Faculty of Veterinary Medicine, Utrecht University were selected for the study. Selection criteria were: a weight range of 10–50 kg, undergone at least one heat, and surgical date 3 months after the last heat. No restriction on age or body condition score was set. Owners completed a questionnaire regarding current health status, last heat cycle, number of previous heat cycles, litters, pseudopregnancies and anti-heat medication prior to the surgery. The study was approved by the institutional ethical committee and all owners provided informed consent for the study.

### Advanced vessel sealers

The new generation LMSD was connected to the ForceTriad energy generator (Medtronic Covidien Valleylab, Eindhoven, The Netherlands) and was used as prescribed: tissue was grabbed, sealed once ensuring that the audible signal of the generator indicated proper seal function, and transected by moving the index finger-controlled cutting blade.

**Fig. 1:**
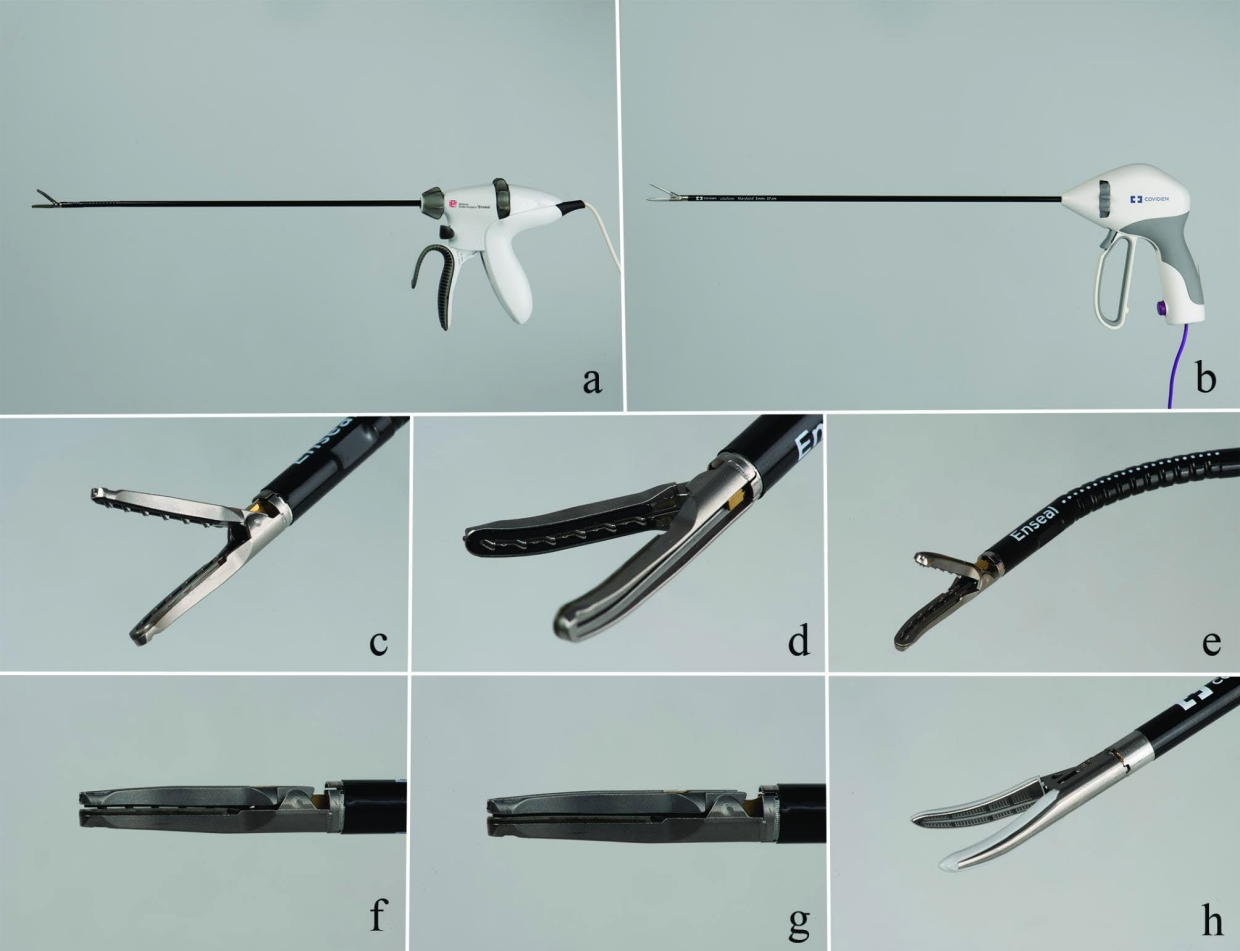
The Enseal G2 (AENG2, **a**, **c-g**) and LigaSure Maryland vessel sealer/divider (LMSD, **b** and **h**). The curved jaw of the ENG2 has multiple small serrations (**c**,**d**) and the vessel sealer provides a 110° articulation perpendicular to the jaw (**e**). While advancing the I-blade, the compression in the tip of the jaw increases (**f**: I-blade not advanced, **g**: I-blade fully advanced). The LVSD has a curved jaw with smooth surface (**h**)

The new-generation AENG2 was connected to the GEN11 power generator (Johnson&Johnson, Amersfoort, The Netherlands) and used as prescribed: tissue was approached via the most ideal orientation using standard rotation function and novel articulation function of the shaft, aiming for orientation of the jaw in line with the desired plane of dissection, perpendicular to the mesovarial blood vessels. Tissue was grabbed and compressed by closing the forceps until the automatic stop, with the handles halfway closed; sealing was commenced by pushing the index finger-controlled button; cutting and continued sealing was performed after the audible signal of the generator changed in frequency by continued closing of the device handles by which the cutting blade was pushed through the tissue; electrical sealing stops when the I-Blade is fully advanced and handles fully closed followed by an audible tone that signals end of sealing event. The AENG2 instruction manual advises to advance the I-blade slowly after the change in beeping tone, especially when transecting larger vessels (Fig. 1).

### Study design

Dogs were randomly assigned to two groups. Prior to surgery, a closed envelope was drawn with a note stating ‘group 1’ or ‘group 2’. In group 1, excision of the left ovary was performed with AENG2 and excision of the right ovary with LMSD. In group 2, the left ovary was excised with LMSD and the right with AENG2.

Anesthesia was performed as per anesthetist preference for ASA 1–2 patients. Local analgesia was performed with lidocaine splash block applied to the ovarian region prior to excision of the ovary in all but 2 patients by penetration of the abdominal wall with a 21G needle.

Patients were clipped between the nipple lines from xiphoid to pubic bone region, placed in dorsal recumbency on a 2-way tiltable operating table supported by a moldable vacuum cushion, followed by aseptic preparation with a chlorhexidine protocol. The patients were draped with disposable surgical drapes. The procedure was performed with a 3-portal midline approach. The patient was placed in 10-15^o^ Trendelenburg position. The abdomen was approached through a modified Hasson technique with a 1 cm skin incision halfway between umbilicus and pubic bone. Subcutis was dissected bluntly and two stay sutures were placed bilateral to the *linea alba* on the external fascial layer of the abdominal wall. The fascial layer was opened with number 11-blade, followed by incision of the internal fascial layer. A 6 mm outer diameter threaded cannula (Ternamian endotip; Karl-Storz-Endoscopy, Amersfoort, the Netherlands) was inserted through the incision. Camera guidance with a 5 mm 0° telescope (Hopkins II, Karl-Storz-Endoscopy) was used to confirm entrance into the abdominal cavity and the abdomen was insufflated with 2-5 L/min CO_2_ and limited at 8 mmHg intra-abdominal pressure (CO_2_ Endo-Arthroflator-Vet, Karl-Storz-Endoscopy). A routine visual exploration of the abdominal cavity was performed. If no abnormalities were noted, the second and third portal were placed in the midline 2-3 cm cranial and caudal to the umbilicus. Both were placed with a 6 mm skin incision, followed by stab incision with an 11-blade under intra-abdominal visual guidance of the camera telescope. Both ovaries were approached and excised in the same standardized manner [5] and left ovariectomy was always performed first [25]. The patient was tilted 20–30° right lateral, towards the surgeon’s side. The round ligament was located near the left inguinal ring and tracked towards the left ovary. The ovarian bursa was grasped and clamped with the lower leg of an atraumatic grasping forceps (Clickline, Karl-Storz-Endoscopy) inside the bursal opening. Excision of the ovarian bursa, containing the ovary, was performed with either AENG2 (group 1) or LMSD (group 2) and was started just caudal of the proper ligament at the uterus tip, extending cranially towards the suspensory ligament. The excised tissue was temporarily placed in the hammock-like pocket formed by the right ventral bladder wall and the ventral bladder ligament while the patient was tilted towards the left-lateral side. Excision of the right ovary was performed in a similar way as described above, using LMSD (group 1) or AENG2 (group 2). The caudal portal was minimally enlarged to facilitate easy removal of the ovaries without damaging the seal. The excision sites were double-checked for absence of bleeding before the remaining CO_2_ was manually pushed out of the abdomen and the cannulas were removed. The *linea alba* was closed with 3 − 0 polydioxanone and the subcutis and dermis with 4 − 0 poliglecaprone-25 sutures.

Intra-abdominal video recordings were made of all excision procedures (Storz Image 1 S 4U; 4 K system, Karl-Storz-Endoscopy). These were assessed by a single person (FD) and scored for the following criteria: fat score of the mesovarium; accessibility of the ovaries, smoke formation, bleeding, abnormality of the uterus and excision times. Fat scores had a range of 0–3: 0 = no; 1 = minimal; 2 = moderate; and 3 = large amount of fat. Accessibility of the ovaries was noted as easy or difficult. Smoke formation was scored from 0 to 2 with 0 = minimal (no impairment of visibility); 1 = moderate (minimal impairment of visibility); 2 = large amount of smoke (moderate impairment of visibility). Bleeding and abnormality of the uterus were noted as present or absent. Excision times were measured from the moment of grasping of uterine tip with the vessel sealer until completed cut of the suspensory ligament. Duration of the surgeries were recorded from time of incision until completed closure. If major bleeding would occur, which could not be stopped with either of the vessels sealers, conversion to an open laparotomy was indicated.

### Device cleaning and sterilization

**Fig. 2:**
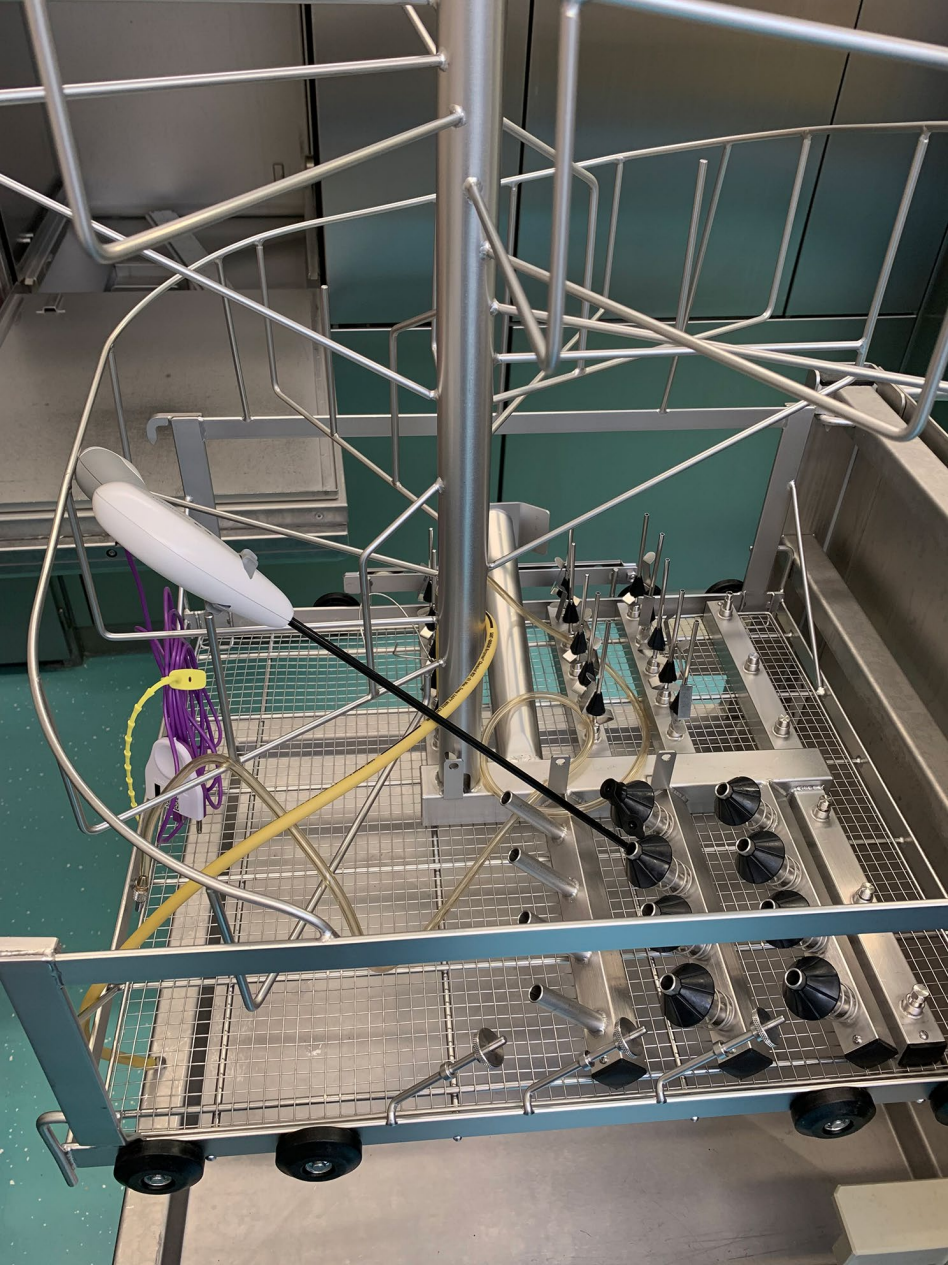
A LigaSure as placed in the rack in the instrument washing machine. The rack is used in human medicine for placement of anesthetic parts, hoses and connectors and slight adjustments have been made for the use of the vessel sealers. Water jets run through the cannulas where the tip of the vessel sealer is placed

AENG2 and LMSD were processed for multiple re-use in our sterilization facility. The vessel sealers were soaked in a tub with a 10% enzymatic cleaning solution (Neodisher MediClean forte, Dr. Weigert, Assen, The Netherlands) for at least 30 min. This was followed by mechanical rinsing with a brush of the contact areas of the jaw. Excessive water and dirt were removed by a high-pressure air blower. Jaws were checked under a stereomicroscope (8 x magnification) for remnants of blood and debris. In case of extreme soiling, an extra step was added with rinsing in an ultrasonic bath with enzymatic cleaning fluid (same as described before). All handpieces were professionally washed in an instrument washing machine (G-7826, Miele, Vianen, The Netherlands) with similar enzymatic cleaning fluid at 60 C in a rack with the tips facing downwards (Fig. 2). Instruments were manually dried and put to rest for a few days to allow evaporation of remnant water. Instruments were marked with a dot on the handpiece to track number of sterilization cycles. The handpieces were double packed and sterilized with ethylene oxide at an external company.

All vessel sealers were used until failure. After termination of the clinical part of the study, remaining AENG2 vessel sealers were sham tested on commercially available chicken breast fillet (8 times per cycle) and processed as above (excluding Ethylene Oxide gas sterilization) until failure of the device occurred.

### Histopathology

Ovaries were collected in 4% neutral-buffered formaldehyde and were processed by routine methods after completion of the clinical trial. The ovaries were sectioned at three sites perpendicular to the seal: at the level of the uterine tip, vascular pedicle and suspensory ligament. The tissues were embedded in paraffin and sectioned at 4 μm and stained with hematoxylin & eosin (Klinipath, Duiven, The Netherlands). Slides were evaluated blindly by a resident in veterinary pathology (JDM) supervised by a specialist in veterinary pathology (GG). Collateral damage was evaluated in the most affected areas and scored quantitatively and qualitatively. For the quantitative analysis, the visible thermal tissue damage was measured in micrometers using an Olympus DP 27 digital camera on a Olympus BX 41 microscope and cellSens entry software (Olympus, Zoetermeer, The Netherlands). For the qualitative analysis, a score of 0–3 was made evaluating coagulation necrosis, loss of cellular detail, formation of an eosinophilic or amphophilic coagulum and nuclear streaming. Any tissue shrinkage during the fixation was not taken into consideration. All slides were evaluated three times and mean qualitative scores were calculated.

### Data collection and statistical analysis

Statistical analysis was performed using IBM SPSS Statistics version 28. A power analysis was performed prior to the study, as described in previous studies [4, 5] (Paired test, β = 0.10; α = 0.05, standard deviation = 25%; estimated difference 20%). A minimum of 19 dogs was needed. Continuous data were assessed for normality with Shapiro-Wilk and Kolmogorov-Smirnov tests. In general data; mean and standard deviation (SD) were calculated for normally distributed data and median and range were calculated for non-normally distributed data. In video scoring and histopathology; continuous data with normal distribution were processed with a paired samples t-test (PSTT). Non-normally distributed data were processed with a Wilcoxon signed-rank test (WSR). Data with ordinal scaling were processed with a Pearson Chi-Square test (PCS) and in case of small numbers a Fisher’s exact test (FE). Significance was considered at P < 0.05 (2-tailed). Both in video scoring and histopathology, data were compared between both left/right and AENG2/LMSD. Effect of fat score and smoke formation on excision times were tested with Kruskal-Wallis (KW) for left, right, AENG2 and LMSD.

## Results

### Patient selection

Twenty-one client-owned dogs that were presented between 28-02-2019 and 02-02-2020 for elective laparoscopic ovariectomy were selected for the study. Median age of the dogs was 21 months (14–83) and mean body weight was 26.5 ± 8.1 kg. The Labrador Retriever was the most common breed (7 dogs), followed by Labradoodle (3), Swiss Shepherd and Malinois (2), Rottweiler, German Shepherd, Cross Breed, Bearded Collie, Golden Retriever, German Shorthaired Pointer, and English Springer Spaniel (one each).

### Surgical Procedure

Videos of 20 dogs were suitable for analyses, the video of one dog had been lost due to technical issues. Total duration for the entire surgical procedure was 63.8 ± 10.9 min. Surgeries were performed by an ECVS diplomate in small animal surgery with extensive experience in advanced laparoscopic surgery (SvN) or by a senior ECVS resident in training with experience in laparoscopic ovariectomies (FD). Closure of portal sites was performed by surgery interns or senior students.

Median mesovarial fat score was 2.0 (1–3). Abdominal and ovarian accessibility was good in all patients. Patient 6 had one abnormally enlarged ovary, but was not taken out of the study as anatomy of the associated mesovarium, proper-, and suspensory ligament appeared to be unaffected. Patient 20 had a slightly enlarged uterus as seen during surgery, ovarian excision was performed as described for inclusion in the study, followed by laparoscopic-assisted hysterectomy.

Smoke formation ranged between 0 and 2 and was not significantly different between left (1.3) and right (1.4) (PCS, P = 0.549), or between AENG2 (1.4) and LMSD (1.2) (PCS, P = 0.535).

Minor bleeding occurred 3 times during ovarian excision in 2 patients: 1 with LMSD during left ovariectomy and 2 with AENG2 during two right ovariectomies. Bleeding could easily be addressed using the same tissue sealer and was not significant between left and right (FE, P = 0.1) and between AENG2 and LMSD (FE, P = 0.1). None of the patients needed conversion to open laparotomy.

**Fig. 3:**
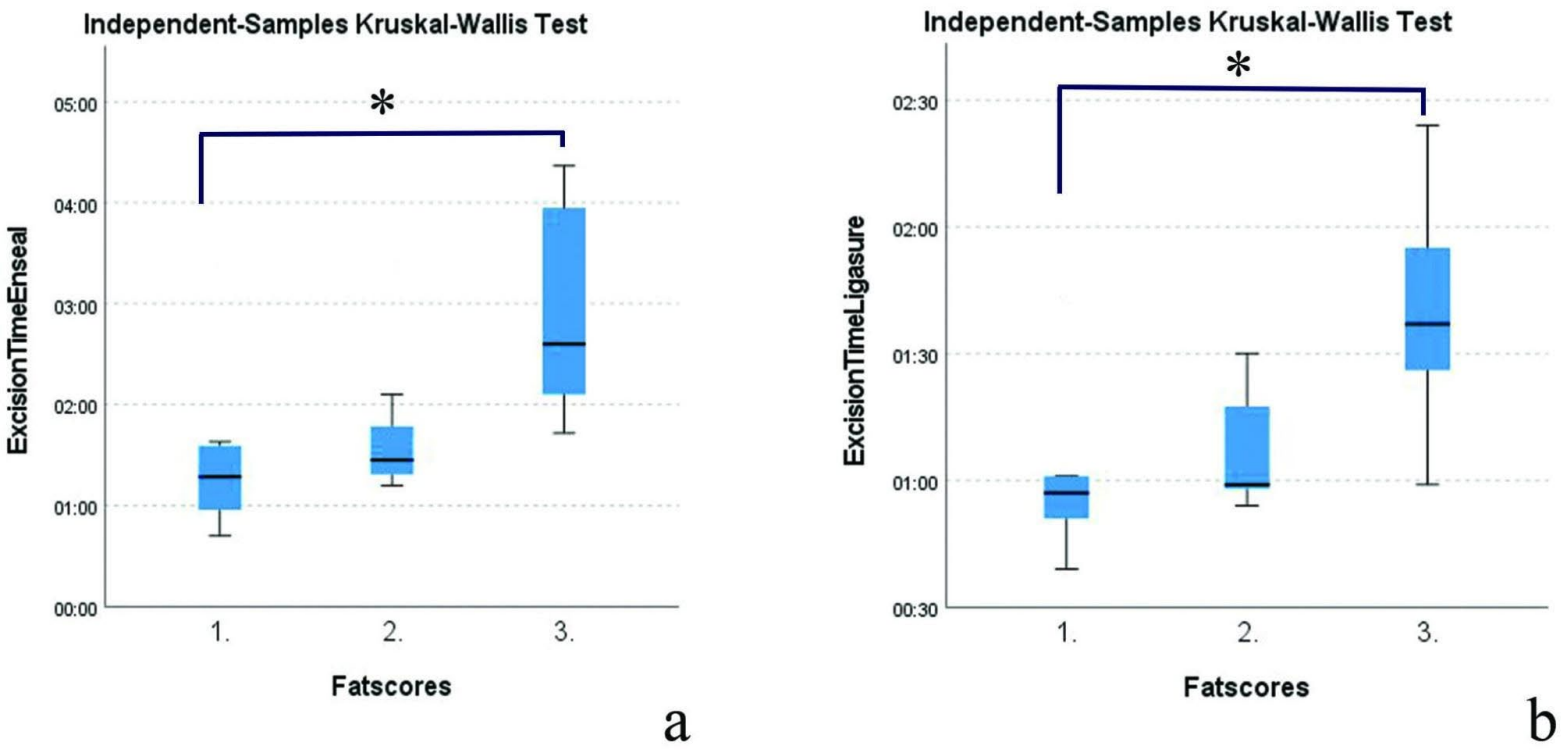
Effect of mesovarian fat score on excision times of AENG2 (**a**) and LMSD (**b**). In both vessel sealers, fat score 3 had a significant longer excision time compared to fat score 1 (asterisk)

Median excision times for the left (01:27 min [00:39 − 03:24]) and right ovaries (01:19 min [00:42 − 04:22]) were not significantly different (WSR P = 0.528). Excision times for AENG2 (01:35 min [00:42 − 04:22]) were significantly longer compared to LMSD (01:00 min [00:39 − 02:24]) (WSR P = 0.001). Excision times with minor bleeding were over median excision times (LMSD: 01:26 min; AENG2 03:57 and 04:22 min). Excision times for both AENG2 (KW P = 0.023) and LMSD (KW P = 0.047) were longer for dogs with a mesovarian fat score of 3 compared to fat score 1. There were no significant differences for both vessel sealers comparing fat score 1 to 2 and 2 to 3 (Fig. 3). Smoke formation did not have any influence on excision times left (KW P = 0.288), right (KW P = 0.505), AENG2 (KW P = 0.864) and LMSD (KW P = 0.494).

Both advanced tissue sealers/dividers were relatively easy to use and caused no handling issues. A major difference in operating these devices lies in the fact that the sealing action of LMSD stops automatically as determined by the system, while the sealing action of AENG2 only stops when the I-Blade is completely advanced, which is user-dependent. The AENG2 instruction manual advises that during the sealing action that is started by pushing the activation button, the I-blade is slowly advanced after the change in beeping tone, especially when transecting larger vessels. The completely device-controlled sealing process of LMSD subjectively led to increased confidence of the operator during tissue transection compared to the partly user-controlled sealing process of AENG2. Subjectively, the use of the articulating function of AENG2 did not improve the accessibility of the ovarian pedicles because when changing the articulation from straight to a bended shaft, either up or down, the angle of approach to the mesovarium became more off-centered from the desired plane of dissection compared to the straight shaft (without angulation). Therefore, added articulation of the shaft was ultimately not applied in any case.

### Sterilization

Re-sterilization of both LMSD and AENG2 was performed as per protocol during the study. Eleven AENG2 vessel sealers were used during this study, with successful re-use after a median of 5 cleaning cycles (range 3–12), device failure occurred one cycle after this. Nine AENG2 broke down due to mechanical failure: falling apart of the body (n = 7) and failure to open jaw (n = 2). Two AENG2 were taken out of use because of electrical failure. Twelve LMSD sealers broke down during the study period. These LMSD devices were re-used after cleaning and re-sterilization up to 9 times, with a mean of 5.3 ± 1.3 cleaning cycles.

### Histopathology

Thirty-eight ovaries of 19 dogs were available for histopathology. Ovaries of patient number 6 were already submitted for histopathology due to a cystic structure on the left ovary that was diagnosed as a teratoma. Ovaries of patient 21 got lost and were consequently not available for histopathology.

On quantitative and qualitative scores, there were no significant difference in collateral thermal tissue damage extent between left and right ovariectomy seals.

**Fig. 4:**
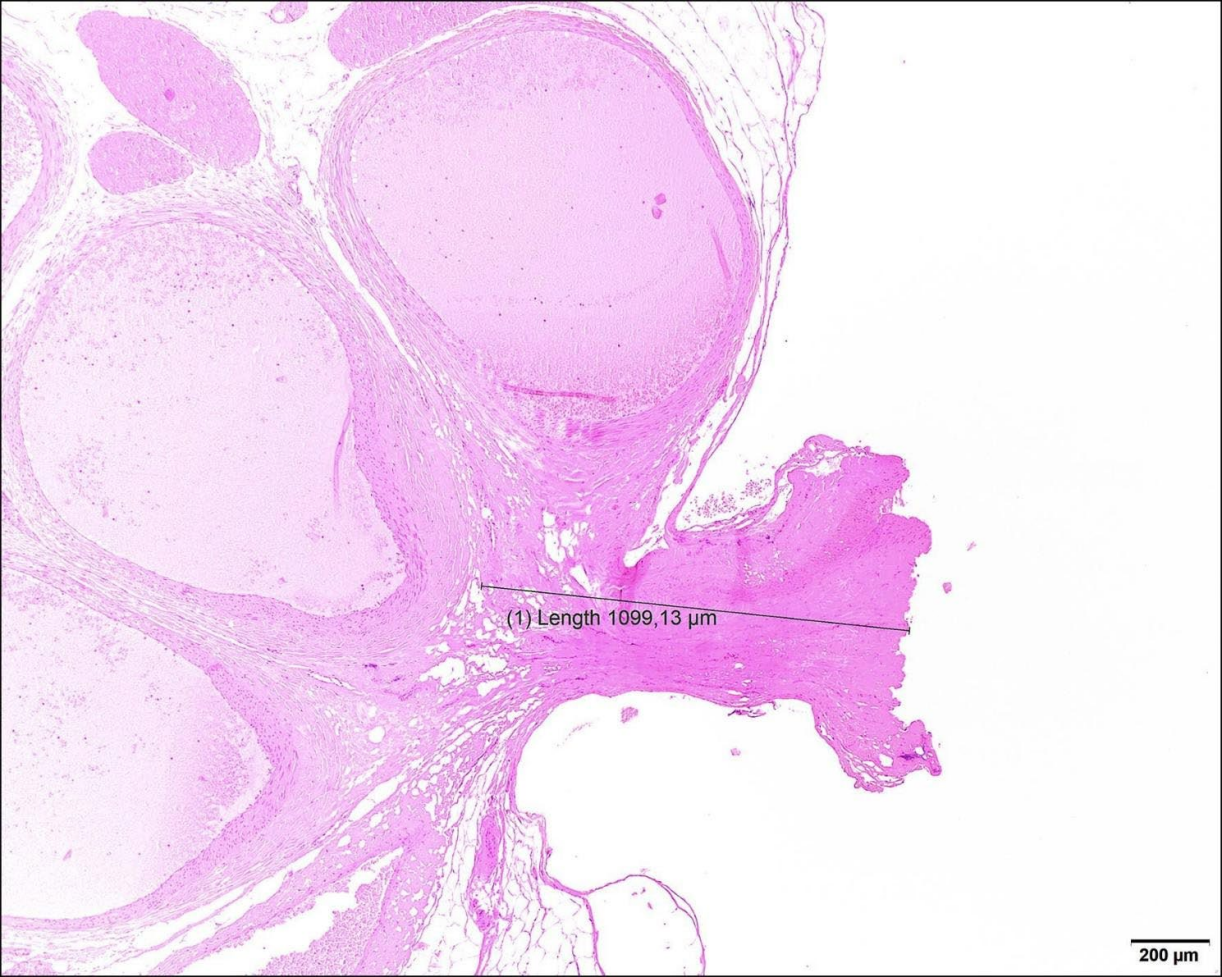
Photomicrograph showing seal at the level of the vascular pedicle. Hematoxylin & eosin staining, obj x4. The completed seal with cut is on the right side of the picture. The black indicator specifies the measured collateral thermal damage in µm

Quantitative tissue damage extent was not significantly different between AENG2 (1.3-2.1 mm) and LMSD (1.0-1.7 mm) for the three sites (Table 1) (Fig. 4). There was no significant difference in qualitative scores on the excised tissue between AENG2 (0.8–1.6) and LMSD (1.0-1.5) (Table 2).

**Table 1:** Histopathology quantitative (maximum collateral damage measured in µm)

	Left	Right	Enseal	Ligasure
Uterine junction	1844.36*	2266.25*	2062.13*	1684.48^#^
	Paired samples t-test P = 0.261		Wilcoxon-signed-rank-test P = 0.494	
Vascular pedicle	1073.49*	1220.70*	1260.05*	1034.14*
	Paired samples t-test P = 0.464		Paired samples t-test P = 0.256	
Suspensory ligament	1960.96*	1756.67*	2042.98*	1674.64*
	Paired samples t-test P = 0.569		Paired samples t-test P = 0.299	

*Normal distributed data, mean. ^#^ Non-normal distributed data, median

**Table 2:** Histopathology qualitative (score 0–3)

	Left	Right	Enseal	Ligasure
Uterine junction	1.53	1.63	1.63	1.53
	Pearson Chi-Square P = 0.670		Pearson Chi-Square P = 0.289	
Vascular pedicle	0.84	0.94	0.83	0.95
	Fisher’s Exact P = 0.167		Fisher’s Exact P = 0.167	
Suspensory ligament	1.00	1.06	1.06	1.00
	Wilcoxon-signed-rank-test P = 0.564		Pearson Chi-Square P = 0.932	

## Discussion

Both bipolar vessel sealers were successfully applied for laparoscopic ovariectomy without difficulties or serious complications. No differences were found between AENG2 and LMSD concerning bleeding or incomplete sealing, smoke formation, and collateral thermal tissue damage. There was a distinct difference in operating these devices. When using LMSD, activation of the sealing mechanism occurs fluently after closure of the forceps through continued squeezing of the handpiece. The power generator indicates by an audible signal that the seal is complete and the electrical sealing action stops automatically, after which the ‘cold’ cutting blade can be advanced using the separate index finger-activated handle. With AENG2, a change in beeping tone of the power generator during sealing indicates that the ‘I-blade’ may be advanced by continued squeezing and closing of the handpiece to its completely closed position while keeping the sealing activation button compressed as the electrical sealing process continues during the advancement of the I-blade, which itself acts as an active electrode. Electrical sealing cycle stops when the I-blade is fully advanced, and thus depends on the speed of transection applied by the user.

Duration of ovary excision was significantly longer with AENG2 compared to LMSD in this study. Excision might be prolonged due to inherent differences in sealing speed between devices and/or to differences in build and operation of the devices, as the Enseal manual does not state at which speed the I-Blade should be advanced, thus leaving room for interpretation by the surgeon. However, considering two minor bleedings with AENG2 in 20 ovary excisions, sealing/transection speed with AENG2 was probably not under-estimated by the surgeon. Another possible cause for prolonged excision duration may be the relative unfamiliarity of both surgeons with AENG2 compared to LMSD. However, AENG2 was used in several ovariectomies and other minimally invasive procedures before the start of the present study in order to get used to operating the device. Furthermore, the results were in agreement with two published studies comparing AENG2 to LMSD, which both show longer excision times for AENG2 versus LMSD in total laparoscopic hysterectomy in humans [12, 14]. Therefore, the difference in ovariectomy duration in the present study is considered a consequence of differences in sealing speed and/or operating features between AENG2 and LMSD.

Excision times were prolonged for both vessel sealers with a higher mesovarial fat score, which is comparable to previous studies in laparoscopic ovariectomy [4, 5].

Minor bleeding occurred three times (2 x AENG2; 1 x LMSD) during ovariectomy. These three incomplete sealing events all occurred in patients with high fat score, and two happened in the same patient (AENG2 and LMSD), indicating that individual patient characteristics may be a risk factor for incomplete tissue sealing rather than bipolar sealing technique. Blood loss was similar between AENG2 and LMSD in total laparoscopic hysterectomy in humans, although a ‘mesovarial fat score’ was not evaluated in humans [12].

Subjectively, the articulating function of the ENG2 was not found to be of added value in 3-portal ovariectomy in this study. The plane of articulation is perpendicular to the opening of the jaw, which does not improve the approach to the uterine tip and mesovarium. Theoretically, the articulation would possibly have been more useful if the articulation had been in the same plane as the opening of the jaw to further improve alignment with the mesovarium. Benefits of the current articulation feature are therefore also questionable for veterinary single-portal ovariectomy, although it has been published to be beneficial in human single site total laparoscopic hysterectomy [26, 27]. A consequence of the use of articulating devices is a significant increase in surgeon-perceived workload, rate of device failure and time to ligation in total laparoscopic hysterectomy in humans [14]. The authors have used the articulating option of the AENG2 in subtotal pericardectomies and have subjectively found the articulation a useful addition in that situation. The usefulness of the articulating feature will depend on specific anatomy and angle of approach and therefore probably differs between specific laparoscopic procedures and approaches and should be further investigated.

Collateral thermal tissue damage was not significantly different between AENG2 and LMSD on quantitative and qualitative analysis in our study. Collateral damage as measured in our study was 1.0-2.2 mm for all sites. These measurements seem comparable to previous studies in which older types of LigaSure and Enseal were compared [5, 6, 15]. It should be noted that histopathologic evaluation of thermal tissue damage extent is not standardized between different studies. Outcomes may be affected by differences in histopathological processing and staining methods, interobserver variability, specific measuring methods (such as in- or exclusion of the tissue seal itself in the measurement of collateral damage extent), tissue composition and thickness, tissue perfusion, and the variability of the energy applied by the device in response to tissue impedance. When comparing results of different studies, it is more reliable to consider relative differences measured under standardized settings within each experiment rather than trying to compare absolute quantitative outcomes of tissue damage extent between different studies.

Both vessel sealers kept functioning after repeatable cleaning and sterilisation cycles as per protocol described in this paper without any negative effects for the patients. However, continuous re-use until failure was not feasible in the present study due to sterilization flow. Multiple AENG2 devices were in rotation to ensure patient flow because gas sterilization by an external company being the limiting factor in turnover time. Consequently, only three ENG2 vessel sealers stopped working by the termination of the patient study. Therefore, remaining AENG2 devices were sham tested until failure after study closure using only our routine cleaning procedure (without Ethylene Oxide gas sterilization). LMSD is a standard tissue sealer in our hospital and multiple devices are in use concurrently, for this study as well as other procedures. Therefore, several of the LMSD devices in the present study may have also been used for other procedures, such as pericardectomy and adrenalectomy. LMSD device failure often showed as an error message in the Force Triad display or loss of the electrical sealing activation knob in the handle, resulting in failure to activate the device; AENG2 device failure presented most often as the breaking apart of the welds of the handpiece after the re-sterilization process. With a median of 5.0 re-uses for AENG2 and a mean of 5.3 re-uses for LMSD, the longevity seems comparable. A previous study by Valenzano et al. [24] mentioned the use of the LMSD for a mean of 7.7 times ± 2.8 times, which seems to be longer compared to our data. However, in that study, devices were not cleaned in an automatic instrument washing machine, which might have negatively affected instrument longevity in our study. This is the second study to report vessel sealers to be successfully cleaned in an instrument washer [5], in that study LMSD was used up to 6–7 times. There are no previous data of repeatable use of Enseal. Subjectively, the authors did not experience any safety issues for the patient with the repeatable use of both vessel sealers; however, we do emphasize on the need to check electrical and mechanical functioning of each device before start of surgery. Further studies about the longevity and safety of AENG2, LMSD, and other vessel sealers after repeatable use with this sterilization protocol are warranted.

## Conclusions

LigaSure Maryland and Articulating Enseal G2 advanced bipolar vessel sealers were similarly effective in laparoscopic ovariectomy in dogs for most tested features. Although AENG2 was significantly slower for ovary excision compared to LMSD, the clinical relevance of this finding in respect of total procedure duration may be debatable. Subjectively, the articulating feature of AENG2 was not of added value in 3-portal ovariectomy in this study and the use of LMSD appeared much more straight-forward and easy because of its completely device-controlled sealing process. The choice of either technique may vary between surgeons, based on individual preference and specific intended use. Both devices were suitable for re-use after repeated cleaning and sterilization cycles, which makes them both interesting for veterinary use.

## Data Availability

The datasets used and/or analyzed during the current study are available from the corresponding author on reasonable request.

## Declarations

### Prior publication

Data included in this article have previously been published in Veterinary Surgery Volume 51, Issue S1: O12-O24 (Abstracts of 2022 Veterinary Endoscopy Society Annual Conference New York, United States of America, June 1–3, 2022).
